# Correlation of pulmonary fat embolism with trauma and resuscitation in children

**DOI:** 10.1007/s00414-025-03516-z

**Published:** 2025-05-23

**Authors:** Rosa Maria Martinez, Wolf Schweitzer, Michael Josef Thali, Akos Dobay, Stephan Andreas Bolliger

**Affiliations:** 1 Repubblica e Cantone Ticino, Institute of Forensic Medicine, Bellinzona, Switzerland; 2 Zurich Institute of Forensic Medicine, Department of Forensic Medicine and Imaging, University of Zurich, Winterthurerstrasse 190/52, CH-8057 Zurich, Switzerland

**Keywords:** Forensic, Pulmonary fat embolism, Resuscitation, CPR, Blunt trauma, Pediatric

## Abstract

**Background:**

This study aims to investigate the correlation between pulmonary fat embolism (PFE), blunt force trauma and the effects of resuscitation in pediatric fatalities.

**Methods:**

Data for this study covered deaths of 57 children aged 0 to 10 years, which underwent full autopsy at the Zurich Institute of Forensic Medicine from 2019 to 2023. Variables collected included anamnestic information on the presence of trauma (accidental, non-accidental), fracture and fatty tissue crushing extent, cardiopulmonary resuscitation (CPR) - with and without intraosseous catheter - and the grade of pulmonary fat embolism (PFE) according to Falzi et al.

**Results:**

The study analyzed 57 pediatric autopsy cases and found that PFE occurred more frequently in cases with both trauma and resuscitation, particularly when intraosseous catheters were used. Fat tissue crushing extent and fracture extent correlated with PFE development, while CPR alone did not. Notably, PFE could arise without fractures, likely due to fatty tissue crushing, and the highest PFE grades were observed in trauma cases with extensive fat crushing.

**Conclusions:**

PFE can arise without fractures, likely from fatty tissue crushing alone. CPR with intraosseous catheters, correlated with moderate PFE (Falzi grade 2), but CPR alone did not strongly predict PFE in children.

## Introduction

Fat embolism, defined as the presence of fat particles in the vascular system, respectively in the pulmonary or peripheral circulation, can occur among other things following various traumas and different medical interventions. Although the exact etiology of pulmonary fat embolism (PFE) is not fully understood, there is a common occurrence in blunt force trauma, with reported incidence rates ranging from 47 to 100% [1–7].

The mechanical theory of pulmonary fat embolism (PFE) development proposed by Gossling et al. [8] proposes that fat particles are liberated into the venous system and become entrapped in the pulmonary capillary bed. From here, they can also migrate to other organs, including the brain and liver, via arteriovenous shunts, leading to the development of a fat embolism syndrome (FES).

FES, first described by Zenker in 1862 and diagnosed by Berger in 1873 [9], must not be confused with PFE. FES typically presents with pulmonary dysfunction and respiratory failure, accompanied by petechiae and fever. It represents a systemic disorder affecting multiple organs and resulting in multi-organ dysfunction [10].

According to prevailing mechanical hypothesis, fat embolism originates from bone fractures and subsequently embolizes into various organs, primarily the lungs [11–13]. It was Falzi et al. [14] who claimed that the severity of PFE correlated with the extent of osseous fractures. Jarmer et al. [15] showed that the fracture grade and the number of body regions affected by fractures correlates significantly with the PFE grade, but not the number of body regions affected by fat crushing and the severity of fat crushing. Falzi et al. additionally reported that the incidence of PFE was 37% in cases with short survival times (minutes), increasing to 60% after several hours and subsequently declining to 42% after several days.

Other research groups have discovered PFE in fatal cases without any fractures and supposed the origin to be fatty tissue crushing [7, 11, 16, and 17]; respectively this being the primary cause of PFE in soft tissue injury and that the number of fractures does not correlate with the grade of PFE [18].

A prospective study of Voisard et al. [19] also concluded that medical interventions like cardiopulmonary resuscitation (CPR) or a combination of trauma and external cardiac massage with rib fractures correlated with PFE [20–22].

While blunt trauma is the most widely postulated etiology of pulmonary fat embolism (PFE), the biochemical theory presumes that humoral alterations induced by trauma or diseases like sepsis lead to the release of chylomicrons into the bloodstream, which subsequently embolize in the lungs.

With his hypothesis, Baker et al. [23] proposed that local hydrolysis of fat emboli by pneumocytes generates free fatty acids. These fatty acids can subsequently distribute to other organs, potentially causing multi-organ dysfunction, thus explaining the presence of fat embolism in rare cases of non-traumatic causes [24]. By occluding the pulmonary vasculature, right heart failure ensues.

Pulmonary fat embolism is important in forensic medicine. On one hand, its presence in some cases shows that the deceased person had an active blood circulation when fat particles entered the blood system and thus constituting a sign of vitality. On the other hand, a severe fat embolism in the pulmonary circulation may be the leading cause of death. Death can result from reduced oxygen uptake, leading to hypoxemia, or due to increased blood pressure in the pulmonary circulation, causing acute, fatal right heart failure [25]. Only persons with healthy hearts can achieve a high grade PFE, as those with pre-existing cardia conditions will succumb to lower PFE grades.

Various correlations between fracture grade and severity of trauma and PFE grade, between PFE and survival time, fat crushing extent, fat crush grade or number of body regions with fractures have already been described in adults [15, 17].

In children, the incidence and severity of pulmonary fat embolism after blunt force trauma and resuscitation is generally considered to be rare [26] and the actual incidence of PFE in pediatric patients remains largely unknown.

This study aims to investigate the correlation between the occurrence of PFE in instances of blunt force trauma including mechanical methods of resuscitation, in order to better understand their associations in children. The ultimate question is whether any identified correlation lends itself to suggest a causal role for the finding of PFE.

## Methods

We retrospectively studied all cases of deceased children between 0 and 10 years of age admitted to the Zurich Institute of Forensic Medicine between 2019 and 2023, which underwent full autopsy. All cases had undergone previous full body CT scanning [27]. Thin sections of lung tissue were collected routinely and examined microscopically with regard to the presence and the extent of pulmonary fat embolism as described earlier [17].

From the autopsy protocols of the 57 children thus included in our study, we examined the following features:

- Age (rounded to the next full year).
- Presence of blunt trauma other than resuscitation.
- Extent of fatty tissue crushing.
- Extent of fractures.
- Presence of mechanical resuscitation.
- Presence of intraosseous catheter (IOC).
- PFE grade according to Falzi et al.
- Case circumstances.
- Survival time (hours)– the estimated time between the collision, fall or other event taking place and ceasing of resuscitation attempts.

As all children were well under puberty, the sex was not considered. Based on the reports, no natural causes for non-traumatic fat embolism could be identified.

For the grading of fracture extent and the fatty tissue crushing extent, we divided the body into head, trunk and extremities and one point was given for each affected body region.

Statistical analysis was performed with the statistical software JMP (SAS Institute, Cary, NC, USA). Significance level was defined as α = 0.05.

## Results

### Trauma cases

Of the 57 cases included, 17 (30%) showed blunt trauma other than CPR. Of these, 13 (23%) received CPR, 8 (14%) with an additional IOC. In the trauma group, ten cases presented a grade 0 PFE, two cases presented a grade 1 PFE, three a grade 2 and two a grade 3 according to Falzi et al. Five of the 17 trauma cases were found to have a grade 0 fat tissue crushing (30%), five a grade 1 (30%), one a grade 2 (5%) and six (35%) suffered an extensive fat crushing (grade 3).

The subgroup trauma without CPR consisted of cases with craniocerebral trauma and drowning after falling from great height and displayed no PFE.

A detailed comparison between the grade of PFE and the subgroups of trauma and CPR (Table 1) shows that CPR as well as blunt trauma correlate with PFE.

**Table 1:** Evaluating the relative role of trauma and CPR, the correlation between presence or absence of trauma and CPR and the average degree of PFE according to falzi is shown.– ^a^Causes of death: isolated craniocerebral trauma (3) and drowning (1)

Cases	Trauma	CPR	PFE grade (mean / SD)
*n* = 6	0	0	0
*n* = 34	0	1	0.3 ± 0.7
*n* = 4^a^	1	0	0
*n* = 13	1	1	1.1 ± 1.2

The data is to be considered sparse with regard to correlating fat crushing and bone injury extent and PFE grade (Table 2).

**Table 2:** Pulmonary fat embolism grade (PFE) compared to the amount of cases per fracture extent grade (FE) and fat crushing extent grade (FCE)

PFE	FE 0	FCE 0	FE 1	FCE 1	FE 2	FCE 2	FE 3	FCE 3
**0**	2	5	6	2	1	1	1	2
**1**	0	0	1	1	1	0	0	1
**2**	1	0	2	2	0	0	0	0
**3**	2	0	0	0	0	0	0	2

Survival time categories (see Table 3) and PFE grade positively correlated with statistical significance ($\:{\chi\:}^{2}$=, Df = 9, 13,8, DF 4, *p* = 0.008).

**Table 3:** Survival time and PFE grades

Survival time	PFE 0	PFE 1 or 2	PFE 3
0 h	6	0	0
0.5–2 h	4	5	1
3 h and more	0	0	1

### Non-trauma cases

Of the 57 cases, 34 cases (58.6%) presented no mechanical trauma other than CPR and of these 15 (25.8%) received an IOC, usually into the tibia.

None of the non-traumatic cases presented resuscitation-associated fractures, such as fractures of the ribs or sternum. All non-traumatic cases with a grade 2 PFE and CPR also received an IOC, whereas only one case with CPR and no IOC presented a PFE, albeit only a grade 1. With an IOC, a grade 2 was observed in 4 out of 15 cases (see Table 4).

**Table 4:** Case counts for CPR and IOC in Non trauma cases by PFE grade

	PFE grade				Total
	0	1	2	3	
No CPR, no IOC	6	0	0	0	6
CPR, no IOC	18	1	0	0	19
CPR and IOC	10	1	4	0	15

### Combined analysis of cases with and without blunt trauma

Across these figures and statistically, the PFE grade correlates more with the extent of fatty tissue crushing and the extent of fractures than with CPR. When correlating the effect that the extent of fractures and fatty tissue crushing (*n* = 17 each) and CPR has on the PFE grade in an ordinal logistic regression (Wald test n.s. for all three factors with *p* ≈ 1 each, as correlate for confirmed proportional odds assumption [28]), the model effects were significant of fat crushing (*p* < 0.0001) and fracture grade (*p* = 0.003) but not for CPR (*p* = 0.16) (Chi-Square test).

## Discussion

This is a small retrospective study comprising only 57 cases. However, we believe that our results permit certain cautious deductions.

According to Ondruschka et al. [21], only mild PFE (grade I) was diagnosed in less than 25% of all cases without trauma after CPR. Our results underline this; sole CPR without IOC led to the development of mild PFE in only one case. However, combined with an IOC, a grade 2 PFE was achieved. This finding is consistent with Castiglioni et al. [22], who noted in their study that the group with CPR and IOC presented a PFE, but the group with only CPR did not.

Therefore, our results imply that PFE due merely to CPR without use of IOC, is not frequent in pediatric fatalities, a finding which stands in stark contrast to adult cases, where PFE due to CPR is seen regularly [19]. A possible explanation for this may be the fact that all our pediatric cases lacked CPR-induced fractures, which is a very frequent finding in adults, especially with increasing age.

Another aspect that this study highlights is that in pediatric fatalities, PFE is observed in cases without skeletal fractures, but by fatty issue crushing alone, a feature ready noted in adult cases [7, 11, 17, 18]. Indeed, the only two cases with a PFE grade 3 in our study collective were children who had suffered extensive non-accidental blunt force trauma with extensive fatty tissue crushing, but no fractures. Survival time correlated with PFE grade.

Statistically, our data showed a significant correlation with the ordinal logistic regression model between the degree of PFE and the extent of fatty tissue crushing and fractures, but not to CPR.

Due to low or absent case counts in various categories that were evaluated, statistical test results have to be interpreted with caution. In particular, hypotheses may be rejected more often due to small sample sizes [29].

## Conclusion

Our results imply that in pediatric fatalities, PFE is not frequent due to mere CPR, additional IOC can, however lead to a moderate PFE even without other mechanical trauma. Furthermore, PFE can arise without fractures and even then seems to be mainly a result of fatty tissue crushing. Furthermore, our study showed a significant correlation of the survival time and the development of PFE.

## Data Availability

The use of anonymized case data for publication was cleared by the Ethics Committee of the Canton of Zurich (15–0686).
